# Supplementary figures and images for: Metabolic responses of wheat seedlings to osmotic stress induced by various osmolytes under iso-osmotic conditions
Source: PLoS One. 2019 Dec 19;14(12):e0226151. doi: 10.1371/journal.pone.0226151 (PMC6922385; doi:10.1371/journal.pone.0226151)

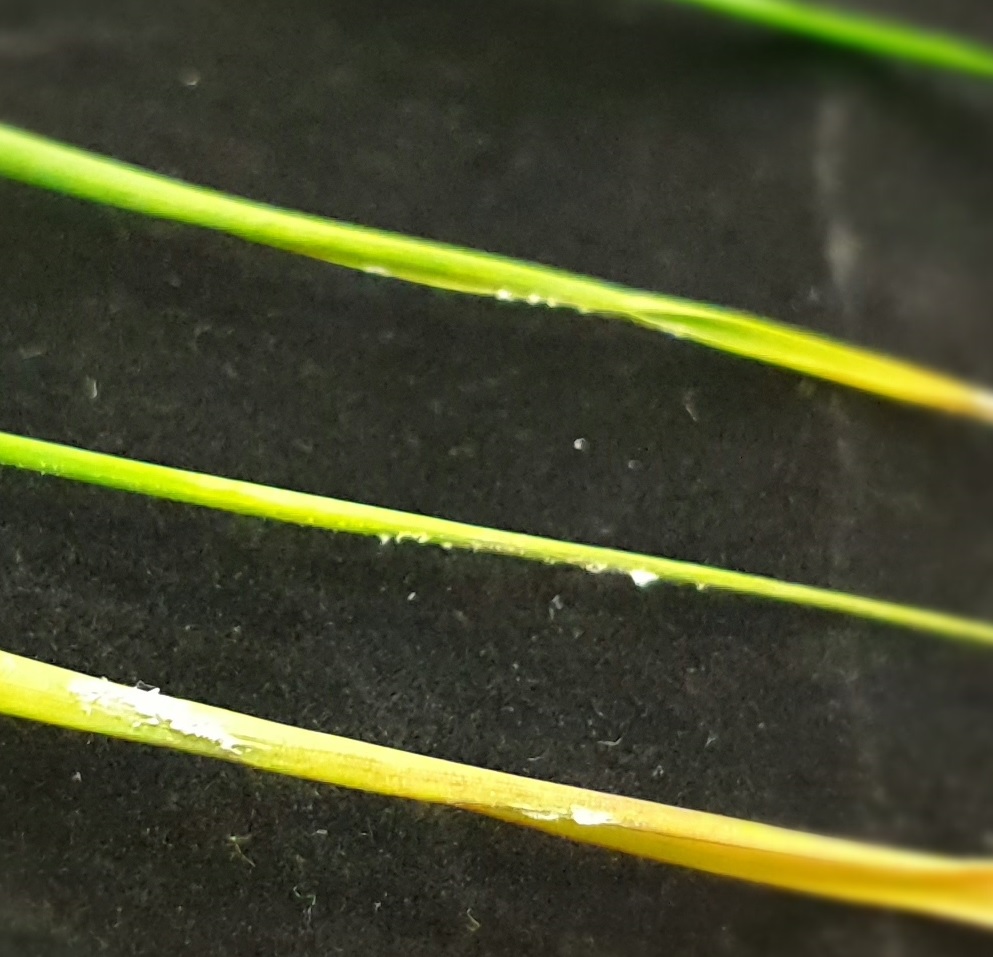

Supplement: S1 Fig — (JPG) [file pone.0226151.s001.jpg]
