# Supplementary material for: Metabolic responses of wheat seedlings to osmotic stress induced by various osmolytes under iso-osmotic conditions
Source: PLoS One. 2019 Dec 19;14(12):e0226151. doi: 10.1371/journal.pone.0226151 (PMC6922385; doi:10.1371/journal.pone.0226151)
Supplement: S1 Table — Different letters indicate significant differences at p < 0.05 level using Tukey’s post hoc test. The results are based on five biological replicates for each treatment and day. (DOCX) [file pone.0226151.s002.docx]

**S1 Table. Results of statistical analysis for the changes of sugar metabolites presented in Figure 6.** Different letters indicate significant differences at the p < 0.05 level using Tukey’s *post hoc* test. The letters indicate a comparison of the days and treatments for the particular sugar component and tissue sap. The results are based on five biological replicates for each treatment and day.

| LEAVES |  | Fructose | Glucose | Sucrose | Maltose | Mannitol | Sorbitol | Galactose |
| --- | --- | --- | --- | --- | --- | --- | --- | --- |
| 2 day | Control | c | f | d | d | c | d | c |
|  | NaCl | g | f | c | d | c | d | c |
|  | PEG | d | b | e | d | c | d | c |
|  | Mannitol | f | f | e | d | b | d | c |
|  | Sorbitol | cd | f | d | c | c | d | c |
|  |  |  |  |  |  |  |  |  |
| 4 day | Control | a | c | d | d | c | d | c |
|  | NaCl | b | de | b | d | c | d | b |
|  | PEG | a | a | d | d | c | d | c |
|  | Mannitol | d | e | de | b | a | d | c |
|  | Sorbitol | b | e | de | b | c | b | c |
|  |  |  |  |  |  |  |  |  |
| 6 day | Control | e | e | d | d | c | d | c |
|  | NaCl | f | f | a | d | c | d | a |
|  | PEG | c | b | c | d | c | d | c |
|  | Mannitol | f | e | c | b | a | d | c |
|  | Sorbitol | d | e | c | a | c | a | c |
|  |  |  |  |  |  |  |  |  |
| ROOT |  | Fructose | Glucose | Sucrose | Maltose | Mannitol | Sorbitol | Galactose |
| 2 day | Control | c | d | e | c | c | d | c |
|  | NaCl | b | c | d | d | c | d | c |
|  | PEG | b | b | c | d | c | d | c |
|  | Mannitol | c | f | c | d | b | d | c |
|  | Sorbitol | b | f | c | b | c | c | c |
|  |  |  |  |  |  |  |  |  |
| 4 day | Control | b | d | e | d | c | d | c |
|  | NaCl | b | e | d | d | c | d | b |
|  | PEG | a | a | b | d | c | d | c |
|  | Mannitol | e | f | e | d | b | d | c |
|  | Sorbitol | e | f | e | d | c | b | c |
|  |  |  |  |  |  |  |  |  |
| 6 day | Control | d | d | cd | d | c | d | c |
|  | NaCl | d | f | a | ab | c | d | a |
|  | PEG | b | b | b | a | c | d | b |
|  | Mannitol | f | f | e | d | a | d | c |
|  | Sorbitol | f | f | e | d | c | a | c |
