# Supplementary material for: Metabolic responses of wheat seedlings to osmotic stress induced by various osmolytes under iso-osmotic conditions
Source: PLoS One. 2019 Dec 19;14(12):e0226151. doi: 10.1371/journal.pone.0226151 (PMC6922385; doi:10.1371/journal.pone.0226151)
Supplement: S2 Table — Different letters indicate significant differences at p < 0.05 level using Tukey’s post hoc test. The results are based on three biological replicates for each treatment and day. (DOCX) [file pone.0226151.s003.docx]

**Results of statistical analysis for free polyamines, putrescine (PUT), spermidine (SPD) and spermine (SPN) determined from the leaves and root, as presented in Figure 8.** The comparison is valid within the lines. Different letters indicate significant differences at p < 0.05 level using Tukey’s *post hoc* test. The results are based on three biological replicates for each treatment and day.

|  |  | Control | NaCl | PEG | Mannitol | Sorbitol |
| --- | --- | --- | --- | --- | --- | --- |
| Leaves | PUT | c | d | c | a | b |
|  | SPD | b | c | b | a | a |
|  | SPN | b | a | b | c | b |
| Root | PUT | b | b | a | a | c |
|  | SPD | b | c | a | d | d |
|  | SPN | b | a | a | c | c |
