# Supplementary material for: Metabolic responses of wheat seedlings to osmotic stress induced by various osmolytes under iso-osmotic conditions
Source: PLoS One. 2019 Dec 19;14(12):e0226151. doi: 10.1371/journal.pone.0226151 (PMC6922385; doi:10.1371/journal.pone.0226151)
Supplement: S3 Table — Data were analysed by using STATISTICA 13.4 software package. (DOCX) [file pone.0226151.s004.docx]

Supplementary Table 3. Analysis variance of various physiological parameters for shoot.

| Source of variation |  | MS | | | | | | | | | |
| --- | --- | --- | --- | --- | --- | --- | --- | --- | --- | --- | --- |
|  | Df | Length | Weight | OP | Proline | GB | Fructose | Glucose | Sucrose | Galactose | Malose |
| Treatment | 4 | 459.6* | 3.3* | 35.7* | 22902.2* | 1857.6* | 917153.9* | 3921925.9* | 53484103.6* | 99192.9* | 1419973.4* |
| Day | 2 | 132.9* | 1.3* | 26.1* | 17688.2* | 2494.0* | 183687.3* | 1675007.9* | 47118997.4* | 45662.9* | 470229.6* |
| Treatment x Day | 8 | 52.5* | 0.5* | 12.2* | 4100.5* | 508.9* | 90948.2* | 71175.7* | 6429929.8* | 45662.9* | 296712.9* |
| Error | 285 | 2.74 | 0.01 | 0.14 | 142.5 | 74.4 | 6097.6 | 6648.6 | 69127.2 | 68.47 | 620 |
| *significant at P ≤ 0.05; 285 (Lengh, weight), 135 (OP =osmotic potential), 60 (proline, GB = glycine betaine) 60 (Fructose, glucose, sucrose, galactose), 58 (maltose) | | | | | | | | | | | |
