# Supplementary material for: Metabolic responses of wheat seedlings to osmotic stress induced by various osmolytes under iso-osmotic conditions
Source: PLoS One. 2019 Dec 19;14(12):e0226151. doi: 10.1371/journal.pone.0226151 (PMC6922385; doi:10.1371/journal.pone.0226151)
Supplement: S4 Table — Data were analysed by using STATISTICA 13.4 software package. (DOCX) [file pone.0226151.s005.docx]

Supplementary Table 4. Analysis variance of various physiological parameters for root.

| Source of variation |  | MS | | | | | | | | | |
| --- | --- | --- | --- | --- | --- | --- | --- | --- | --- | --- | --- |
|  | Df | Length | Weight | OP | Proline | GB | Fructose | Glucose | Sucrose | Galactose | Malose |
| Treatment | 4 | 250.5* | 1.7* | 257.1* | 22902.2* | 2464506.7* | 2199725.4* | 3901012.7* | 7307021.9* | 12729798.9* | 31495727.8* |
| Day | 2 | 10.5* | 0.2* | 0.2* | 4.3 | 2165.5 | 1829414.5* | 262674.4* | 3009015.7* | 20817894.9* | 38543761.2* |
| Treatment x Day | 8 | 14.3* | 0.2* | 0.2* | 17.5* | 993.2 | 503037.4* | 261033.1* | 4184319.7* | 13407486.1* | 21887840.1* |
| Error | 285 | 133.6 | 0.87 | 0.00 | 0.01 | 3733.8 | 21508 | 5530 | 13888 | 8320 | 20988 |
| *significant at P ≤ 0.05; 285 (Lengh, weight), 133 (OP =osmotic potential), 60 (proline, GB = glycine betaine), 60 (Fructose), 58 (glucose, galactose), 75 (sucrose),  58 (maltose) | | | | | | | | | | | |
