# Supplementary material for: Metabolic responses of wheat seedlings to osmotic stress induced by various osmolytes under iso-osmotic conditions
Source: PLoS One. 2019 Dec 19;14(12):e0226151. doi: 10.1371/journal.pone.0226151 (PMC6922385; doi:10.1371/journal.pone.0226151)
Supplement: S5 Table — Principle component analysis (PCA) was applied for evaluation of response of shoots under control and osmotic stresses. Data were analysed by using STATISTICA 13.4 software package. (DOCX) [file pone.0226151.s006.docx]

**Supplementary Table 5.** **Factor loadings of sugars, proline, GB and osmotic potential.** Principle component analysis (PCA) was applied for evaluation of response of shoots under control and osmotic stresses. Data were analysed by using STATISTICA 13.4 software package.

| Variable | PC1 | PC2 | PC3 | PC4 |
| --- | --- | --- | --- | --- |
| OP | -0.80 | -0.44 | 0.23 | 0.25 |
| Proline | 0.94 | -0.17 | -0.19 | -0.15 |
| GB | 0.22 | 0.00 | 0.86 | 0.16 |
| Fru | -0.57 | 0.17 | -0.66 | -0.18 |
| Glu | -0.54 | -0.01 | -0.54 | -0.38 |
| Suc | 0.70 | -0.56 | -0.38 | 0.06 |
| Gal | 0.64 | -0.66 | -0.33 | 0.07 |
| Maltose | 0.47 | 0.85 | -0.13 | 0.09 |
| Mannitol | 0.35 | 0.31 | 0.41 | -0.77 |
| Sorbitol | 0.29 | 0.74 | -0.37 | 0.47 |
| Explained variance (eigenvalue) | 3.52 | 2.36 | 2.13 | 1.11 |
| Proportion of total variance (%) | 35.17 | 23.60 | 21.34 | 11.10 |
| Cumulative variance (%) | 35.17 | 58.76 | 80.10 | 91.20 |

PC-1 (principal component 1); PC-2 (principal component 2); PC-3 (principal component 3)
