# Supplementary material for: Metabolic responses of wheat seedlings to osmotic stress induced by various osmolytes under iso-osmotic conditions
Source: PLoS One. 2019 Dec 19;14(12):e0226151. doi: 10.1371/journal.pone.0226151 (PMC6922385; doi:10.1371/journal.pone.0226151)
Supplement: S6 Table — Principle component analysis (PCA) was applied for evaluation of response of roots under control and osmotic stresses. Data were analysed by using STATISTICA 13.4 software package. (DOCX) [file pone.0226151.s007.docx]

| Variable | PC1 | PC2 | PC3 |
| --- | --- | --- | --- |
| OP | 0.73 | 0.18 | 0.20 |
| Proline | -0.77 | -0.41 | 0.04 |
| GB | -0.37 | 0.79 | -0.06 |
| Fru | -0.42 | 0.78 | 0.08 |
| Glu | -0.24 | 0.94 | -0.02 |
| Suc | -0.95 | -0.03 | -0.04 |
| gal | -0.86 | -0.36 | -0.02 |
| maltose | -0.83 | -0.34 | 0.11 |
| mannitol | 0.35 | -0.34 | -0.81 |
| Sorbitol | 0.28 | -0.35 | 0.73 |
| Explained variance (eigenvalue) | 3.815 | 2.553 | 1.310 |
| Proportion of total variance (%) | 38.147 | 25.532 | 13.104 |
| Cumulative variance (%) | 38.147 | 63.679 | 76.783 |

**S Table 6.** **Factor loadings of sugars, proline, GB and osmotic potential.** Principle component analysis (PCA) was applied for evaluation of response of roots under control and osmotic stresses. Data were analysed by using STATISTICA 13.4 software package.

PC-1 (principal component 1); PC-2 (principal component 2); PC-3 (principal component 3)
